# Flying lemurs – The 'flying tree shrews'? Molecular cytogenetic evidence for a Scandentia-Dermoptera sister clade

**DOI:** 10.1186/1741-7007-6-18

**Published:** 2008-05-01

**Authors:** Wenhui Nie, Beiyuan Fu, Patricia CM O'Brien, Jinhuan Wang, Weiting Su, Alongkoad Tanomtong, Vitaly Volobouev, Malcolm A Ferguson-Smith, Fengtang Yang

**Affiliations:** 1 State Key Laboratory of Genetic Resources and Evolution, Kunming Institute of Zoology, Chinese Academy of Sciences, Kunming, Yunnan 650223, People's Republic of China; 2 Cambridge Resource Centre for Comparative Genomics, Department of Veterinary Medicine, University of Cambridge, Madingley Road, Cambridge CB3 0ES, UK; 3 Department of Biology, Faculty of Science, Khon Kaen University, Khon Kaen 40002, Thailand; 4 Muséum National d'Histoire Naturelle, Origine, Structure et Evolution de la Biodiversité, rue Buffon, 75005 Paris, France; 5 Wellcome Trust Sanger Institute, Wellcome Trust Genome Campus, Hinxton, Cambridge CB10 1SA, UK

## Abstract

**Background:**

Flying lemurs or Colugos (order Dermoptera) represent an ancient mammalian lineage that contains only two extant species. Although molecular evidence strongly supports that the orders Dermoptera, Scandentia, Lagomorpha, Rodentia and Primates form a superordinal clade called Supraprimates (or Euarchontoglires), the phylogenetic placement of Dermoptera within Supraprimates remains ambiguous.

**Results:**

To search for cytogenetic signatures that could help to clarify the evolutionary affinities within this superordinal group, we have established a genome-wide comparative map between human and the Malayan flying lemur (*Galeopterus variegatus*) by reciprocal chromosome painting using both human and *G. variegatus *chromosome-specific probes. The 22 human autosomal paints and the X chromosome paint defined 44 homologous segments in the *G. variegatus *genome. A putative inversion on GVA 11 was revealed by the hybridization patterns of human chromosome probes 16 and 19. Fifteen associations of human chromosome segments (HSA) were detected in the *G. variegatus *genome: HSA1/3, 1/10, 2/21, 3/21, 4/8, 4/18, 7/15, 7/16, 7/19, 10/16, 12/22 (twice), 14/15, 16/19 (twice). Reverse painting of *G. variegatus *chromosome-specific paints onto human chromosomes confirmed the above results, and defined the origin of the homologous human chromosomal segments in these associations. In total, *G. variegatus *paints revealed 49 homologous chromosomal segments in the HSA genome.

**Conclusion:**

Comparative analysis of our map with published maps from representative species of other placental orders, including Scandentia, Primates, Lagomorpha and Rodentia, suggests a signature rearrangement (HSA2q/21 association) that links Scandentia and Dermoptera to one sister clade. Our results thus provide new evidence for the hypothesis that Scandentia and Dermoptera have a closer phylogenetic relationship to each other than either of them has to Primates.

## Background

Extensive research, based on large data sets of amino acid, nuclear and mitochondrial sequences from broad mammalian taxonomic representatives, has led to a new consensus on the phylogenetic relationships of the 18 extant placental orders. Such studies have identified four superordinal clades: Afrotheria (Proboscidea, Hyracoidea, Sirenia, Tubulidentata, Macroscelidea and Arosoricida), Xenarthra (sloths, anteaters and armadillos), Supraprimates or Euarchontoglires (Rodentia, Lagomorpha, Primates, Dermoptera and Scandentia) and Laurasiatheria (Cetartiodactyla, Perissodactyla, Carnivora, Pholidota, Chiroptera and Eulipotyphla) [1-8]. However, the phylogenetic relationships among the members within each of the four superordinal clades are not well-resolved [9]. For example, a well-known case is the evolutionary affiliations of Euarchonta (= Primates + Dermoptera + Scandentia) [1], a subgroup within Supraprimates [2] (also called Euarchontoglires = Euarchonta + Glires (Rodentia + Lagomorpha)) [1,4].

Within Euarchonta, the relationship between Primates, Dermoptera and Scandentia remains unresolved [8,10-12] and various hypotheses on the inter-ordinal relationships have been proposed by different morphological and molecular phylogenetic studies. In fact, all possible combinations have been proposed, including: (a) a closer relationship between Scandentia and Primates [13-15]; (b) a closer relationship between Primates and Dermoptera [1,2,8,16-19], forming the clade Primatomorpha [18]; (c) Dermoptera as a sister group to the anthropoid primates [3,20]; (d) Dermoptera as the closest living relative of Primates [21]; (e) a closer relationship between Scandentia and Dermoptera [4-7,22]. Furthermore, based on some intriguing similarities in morphology and molecular data, a sister-group relationship between tree shrews (Scandentia) and lagomorphs (Lagomorpha) has also been proposed by several studies [15,23,24]. Nevertheless, molecular and morphological studies so far have not unambiguously resolved the relationships among Primates, Dermoptera and Scandentia, suggesting a need to search for other evidence such as shared signatures provided by chromosomal rearrangements.

Cross-species chromosome painting [25,26] is a powerful method for investigating the evolution of genome organizations. This method enables (1) the rapid and reliable identification of homologous chromosome segments between any two species in placental mammals based on DNA sequence homology, (2) the tracking of chromosomal rearrangements which have occurred during evolution based on the distribution pattern of conserved chromosome segments, and (3) an independent verification of the molecular phylogenetic tree using signature chromosomal rearrangements [27-34]. Chromosomal homologies between human and representative species of other placental orders have been established by cross-species chromosome painting using human chromosome-specific probes. Up to now, only two (Dermoptera and Hyracoidea) of the 18 extant placental orders have no published genome-wide comparative maps with human. While data from these orders are critical for understanding the evolution of genome organization of placental mammals as a whole, data from Dermoptera are also pivotal for the reconstruction of the ancestral karyotype and evolutionary history of all primates.

Here, for the first time, we have established the genome-wide chromosomal correspondence between human and the Malayan flying lemur (*Galeopterus variegatus*, GVA, 2*n *= 56), one of the two living species in Dermoptera, by reciprocal cross-species chromosome painting using human and GVA probes. Comparative analysis of available comparative chromosome maps of representative species of Supraprimates has revealed cytogenetic evidence that unites the Scandentia and Dermoptera into one clade.

## Results

### The G-banded karyotype and flow karyotype of *G. variegatus*

**Figure 1:**
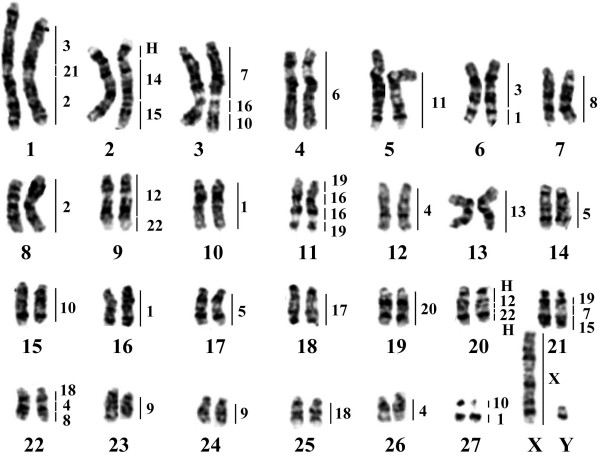
**G-banded karyotype of *G. variegatus *(2*n *= 56, GVA), with a summary of the chromosome painting with human paints**. Chromosome numbers of GVA are indicated below the chromosomes and the segments homologous to human are indicated to the right of each chromosome. H: heterochromatin.

As reported previously [35], *G. variegatus *has a 2*n *= 56 karyotype consisting of eight pairs of bi-armed (# 2, 4–6, 8, 11, 13 and 27) and 19 pairs of acrocentric autosomes (Figure 1). Chromosome 27 has a secondary constriction, the X chromosome is a large metacentric chromosome, and the Y chromosome is the smallest bi-armed chromosome. The G-banded karyotype of *G. variegatus *has not been reported previously.

**Figure 2:**
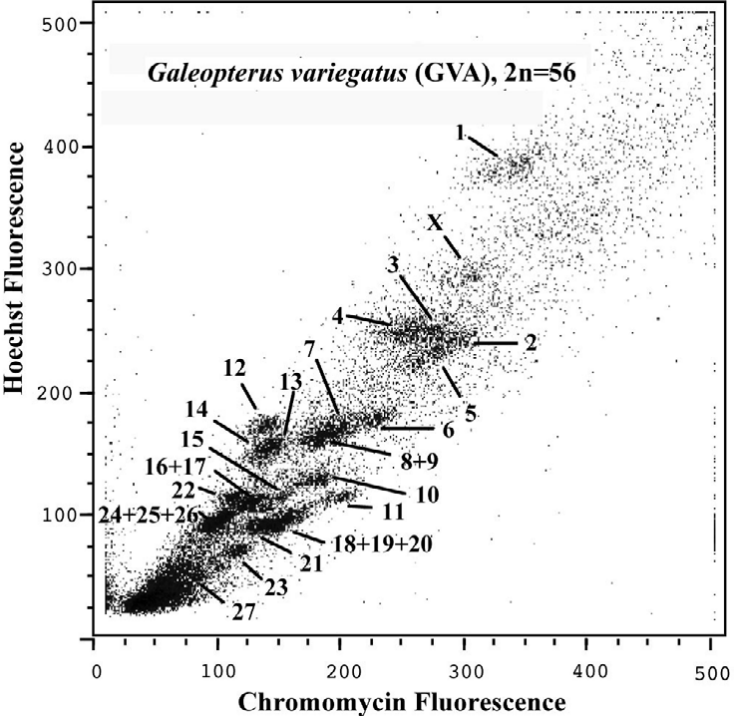
Bivariate flow karyotype of *G. variegatus *with chromosome assignments.

The chromosomes of *G. variegatus *were resolved into 22 separate regions (Figure 2). Chromosome paints prepared from individual regions were hybridized to metaphases of *G. variegatus*, allowing the identification of chromosomes contained in each region. Except for four regions, all other regions each contained only one type of *G. variegatus *chromosomes. Of these four regions, two regions each contained two *G. variegatus *chromosomes (GVA8+9 and 16+17), the other two regions each contained three *G. variegatus *chromosomes (GVA18+19+20 and 24+25+26). A complete set of *G. variegatus *chromosome-specific paints except for the Y was generated.

### Reciprocal painting between *G. variegatus *and human

**Figure 3:**
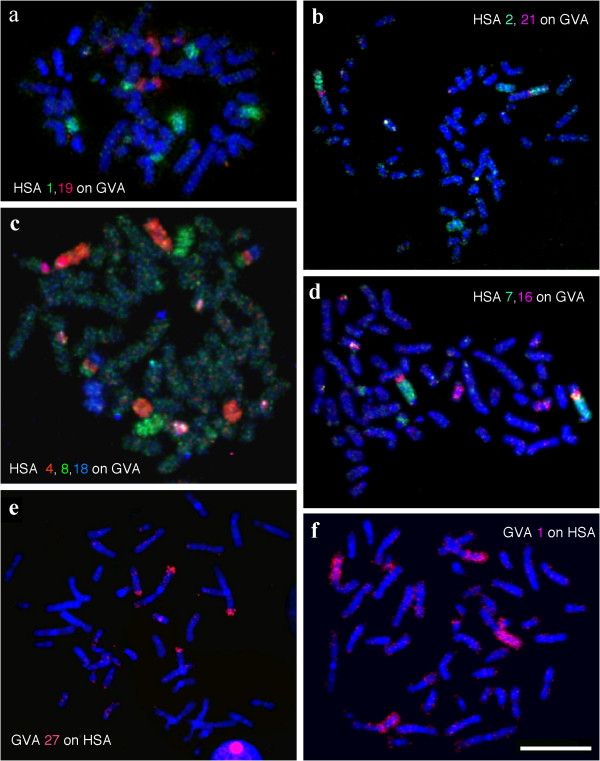
**Examples of reciprocal chromosome painting of human (HSA) probes on *G. variegatus *(GVA) metaphases and *G. variegatus *paints on human metaphases**. (a)–(d) Human (HSA) probes on *G. variegatus *(GVA) metaphases; (e), (f) *G. variegatus *paints on human metaphases.

To establish the genome-wide chromosomal correspondence between *G. variegatus *and human, the 22 human autosomal probes and the X probe were firstly hybridized onto *G. variegatus *chromosomes. Fluorescent *in situ *hybridization (FISH) examples are presented in Figure 3. The hybridization results are summarized onto a *G. variegatus *G-banded karyotype (Figure 1). Eight human chromosome probes (HSA6, 11, 13, 14, 17, 20, 21 and X) each painted one *G. variegatus *chromosomal segment or chromosome. Painting probes derived from 10 human chromosomes (HSA2, 3, 5, 7–9, 12, 15, 18 and 22) each hybridized onto two different *G. variegatus *chromosomes. Four human chromosome probes (HSA4, 10, 16, 19) each delimited three homologous *G. variegatus *chromosomal segments. Human chromosome probe 1 gave four signals on four *G. variegatus *chromosomes. The heterochromatic regions of *G. variegatus*, such as the centromeric regions of most chromosomes, the short arm of GVA2 and two parts of GVA20, were not painted by any human chromosome probe. Together, 22 human autosomal paints and the X chromosome paint defined 44 homologous segments in the *G. variegatus *genome. The hybridization patterns of human chromosome probes 16 and 19 revealed a putative inversion on GVA11. Fifteen combinations of adjacent human homologous segments, HSA1/3, 1/10, 2/21, 3/21, 4/8, 4/18, 7/15, 7/16, 7/19, 10/16, 12/22 (twice), 14/15 and 16/19 (twice), were detected in the *G. variegatus *genome.

**Figure 4:**
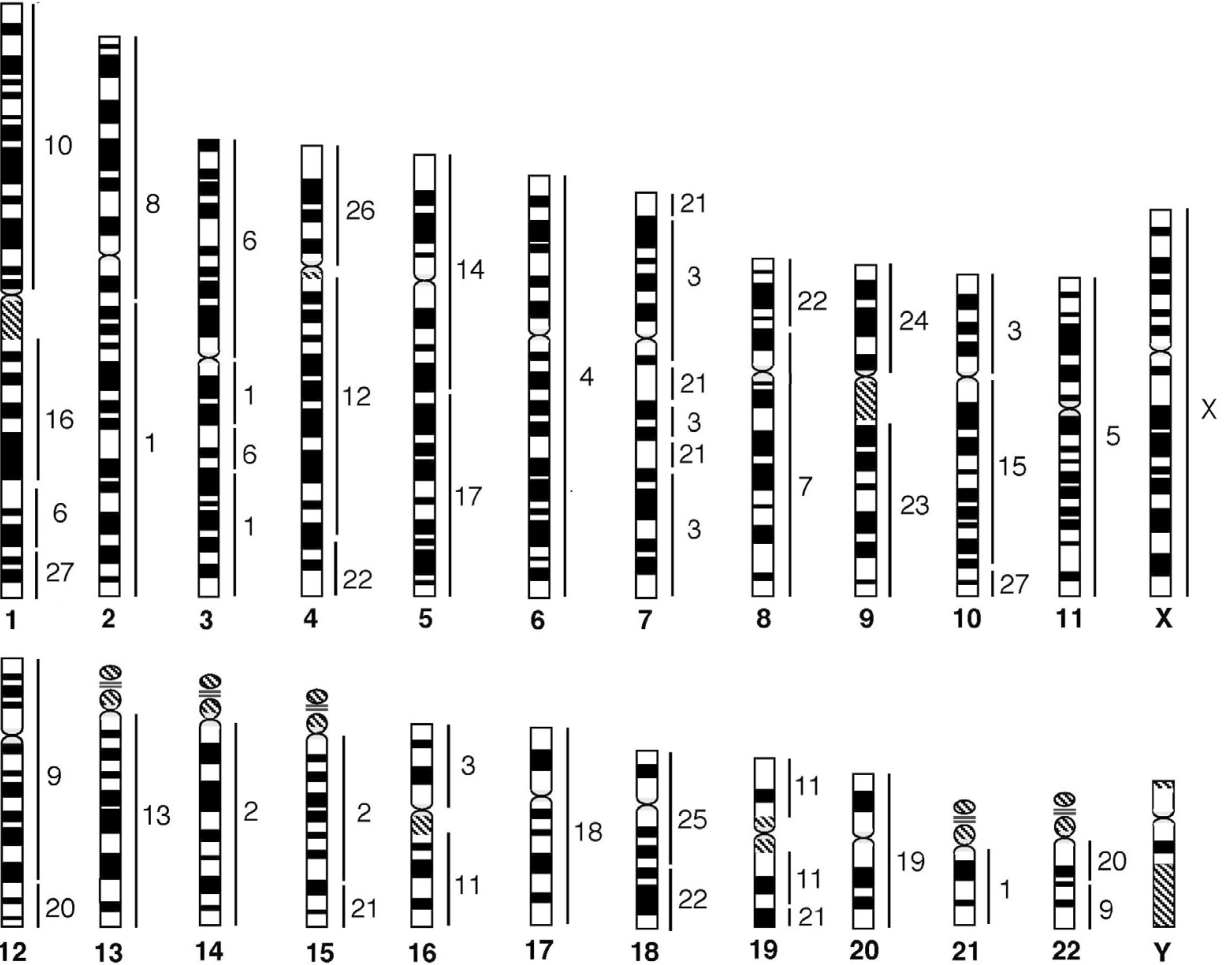
**Summary of the chromosome painting with *G. variegatus *probes onto human on a G-banded idiogram**. Human chromosome numbers are indicated below the chromosomes and the segments homologous to *G. variegatus *are indicated to the right of each chromosome.

To further verify these results, and to define the origin of the homologous human chromosomal segments in the segment associations detected in the *G. variegatus *genome, we carried out reverse painting of *G. variegatus *chromosome-specific paints onto human chromosomes. Examples are shown in Figure 3, and the results are summarized against an idiogram of the G-banded HSA karyotype (Figure 4). Eighteen *G. variegatus *paints (GVA4, 5, 7, 8, 10, 12–19, 23–26 and X) each labeled one HSA chromosome or chromosomal segment. Four paints (GVA2, 9, 20 and 27) each hybridized to two HSA chromosomal segments. Three paints (GVA6, 11 and 22) each painted three HSA chromosomal segments. GVA paint 1 gave hybridization signals on four HSA chromosomal segments, and GVA paints 3 and 21 each produced hybridization signals on five HSA chromosomal segments. In total, *G. variegatus *paints revealed 49 homologous chromosomal segments in the HSA genome.

## Discussion

Resolving the phylogenetic placement of Dermoptera within Euarchonta is of fundamental importance to our understanding of the origin and evolution of human genome organization. The establishment of a genome-wide comparative chromosome map between human and *G. variegatus*, filled our knowledge gap on the comparative genome organization of Dermoptera, and ensured that each order contained in the Euarchonta group has at least one representative species for which a genome-wide comparative map with human is available. An integrated analysis of the distribution patterns of synteny-conserved homologous segments in Dermoptera and in the representative species of Primates, Scandentia, Rodentia and Lagomorpha, enabled us to search for signature rearrangements that might provide further insight into the phylogenetic placement of Dermoptera within Euarchonta and Supraprimates.

It has been demonstrated that chromosomal rearrangements (including shared derived chromosomal syntenic associations revealed through chromosome painting) can serve as valuable phylogenetic markers to interpret the relationships of the 18 extant placental orders by the principle of parsimony [27,29,31-34,36-40]. When a derived chromosomal association (segments that are syntenic to two or more human chromosomes found on a single chromosome in another species) is shared by various species, this particular chromosome character (that is, a synapomorphy) may reflect a common evolutionary origin or a close phylogenetic relationship of these species [27,28,33]. For instance, the HSA5/21 and 1/19p in Afrotheria [31,32,39], and HSA7q/10p and 2/8 in Xenarthra [40], are considered as signature rearrangements for the superordinal clades Afrotheria and Xenarthra, respectively.

In Primates, over 70 species, including several prosimian species, have been analyzed so far by chromosome painting with human chromosome-specific probes. Except for five common ancient associations, HSA3/21, 7/16, 12/22 (twice) and 14/15, which are ancestral for all placental mammals, various landmark rearrangements have been identified for most of the nodes in primate phylogeny [41-47]. Such landmark rearrangements include HSA2/4, 4/6 and 8/15 associations in Lemuriform prosimians [45], HSA1q/19p, 2/12/22, 6/14, 12/7/16, 9/15 and 10/19q associations in Lorisiform prosimians [42,47], and HSA5/7, 8/18 and 10/16 in the New World monkeys [46]. In the great apes and Old World monkeys, chromosome-painting reveals that their karyotypes are highly conserved, only a few chromosomal changes differentiate their karyotypes from the ancestral karyotype of all primates [46]. However, the karyotypes of the lesser apes or gibbons dramatically differ from those of other primates in having an exceptionally high rate of chromosomal rearrangement [43,48-52].

In Scandentia, the chromosomal homologies have been defined between human and a tree shrew (*Tupaia belangeri*, TBE, 2*n *= 62) by reciprocal chromosome painting [53]. Results indicated that there are three derived associations (HSA2/21, 10/16 and 11/20) retained in the *T. belangeri *genome. However, a subsequent study by Richard et al revealed the existence of a HSA7 homologous segment (HSA7b), which forms part of the ancestral placental syntenic association of HSA7b/16p, between HSA10 and 16 segments on TBE chromosome 1p [54]. Thus, the HSA10/16 association should be considered as absent in the *T. belangeri *genome. Instead, the association of HSA7b/10p should be considered as an additional derived character for *T. belangeri*. In Dermoptera, our chromosome painting results revealed eight additional derived human chromosomal segment associations (HSA1/3, 1/10, 2/21, 4/18, 7/15, 7/16p, 7/19, 10p/16p) in the genome of *G. variegatus*. Our reciprocal chromosome painting results shows that the HSA7/16 association found in *G. variegatus *is not the same as the ancestral HSA7b/16p syntenic association found in most placental mammals. The ancestral HSA7b/16p syntenic association has broken into two chromosomal segments in *G. variegatus *(GVA3 and 21). In Lagomorpha, the chromosome map between human and the rabbit (*Oryctolagus cuniculus*) has also been established by reciprocal chromosome painting [55]. In Rodentia, chromosome painting results have demonstrated that murid rodents have highly rearranged genomes [56,57], while the karyotypes of squirrels are highly conserved [58-60]. Two shared derived syntenic associations between the rabbit and squirrels (HSA1/10 and 9/11) [60] have provided additional support for the clade Glires (Lagomorpha + Rodentia) [1].

We have compared the above chromosome painting data of different representative species of each order within the Supraprimates. However, we failed to detect any signature rearrangement that would support a close phylogenetic relationship either between *G. variegatus *and Primates or between Dermoptera and Glires. Although the *G. variegatus *and the New World monkeys share the one derived association of HSA10/16, while *G. variegatus*, rabbits and squirrels share the HSA1/10 association, the reverse painting results indicate that the two associations detected in *G. variegatus *and the other species had different origins. The segments contained in the HSA10/16 association of *G. variegatus *originated from HSA10p and 16p, while in the New World monkeys, they were derived from HSA10q and 16p [61]. In the genome of *G. variegatus*, the segments contained in HSA1/10 association are homologous to HSA1q and10q (Figure 4), while in the genomes of rabbits and squirrels, they are homologous to HSA1q and10p [55,60]. Furthermore, comparison of the chromosome map between human and rabbits [55] with the map between human and *T. belangeri *[53] reveals no shared derived human chromosomal association that unites Scandentia and Lagomorpha into one group either.

The most salient finding comes from the comparison of the comparative chromosome map between human and *G. variegatus *with the map between human and *T. belangeri*. Such a comparison revealed one common derived human syntenic segment association, HSA2q/21, in both *T. belangeri *and *G. variegatus*. The results of reverse painting using the probes of *T. belangeri *and *G. variegatus *to hybridize human chromosomes demonstrated that HSA2q/21 association in both *T. belangeri *and *G. variegatus *originated from the same human homologous segments: HSA2q and 21(Figure 4 in this study, and Figure 5 in [53]). This syntenic association has not been detected in any other mammal so far studied by chromosome painting, including bats (order Chiroptera). We thus believe that the HSA2/21 association most likely represents the signature rearrangement that unites Scandentia and Dermoptera orders into one sister clade. Such a clade has been proposed previously by several molecular studies but with moderate to weak bootstrap support [4-7,22]. This grouping is in sharp contrast to a most recent molecular study that suggested the flying lemur as the closest living relative of primates [21]. It should be mentioned that so far only one representative species from each of Scandentia and Dermoptera has been studied by chromosome painting. Most species in Scandentia have a karyotype 2*n *= 52–68, with only one species (*Urogale everetti*) having 2*n *= 44 [62], while in Dermoptera there is the species (*Cynocephalus volans*) with 2*n *= 38 [63]. Further chromosome painting between human and these low diploid number species will help to validate if the HSA2q/21 association is indeed the true signature rearrangement that link Scandentia and Dermoptera into one clade.

**Figure 5:**
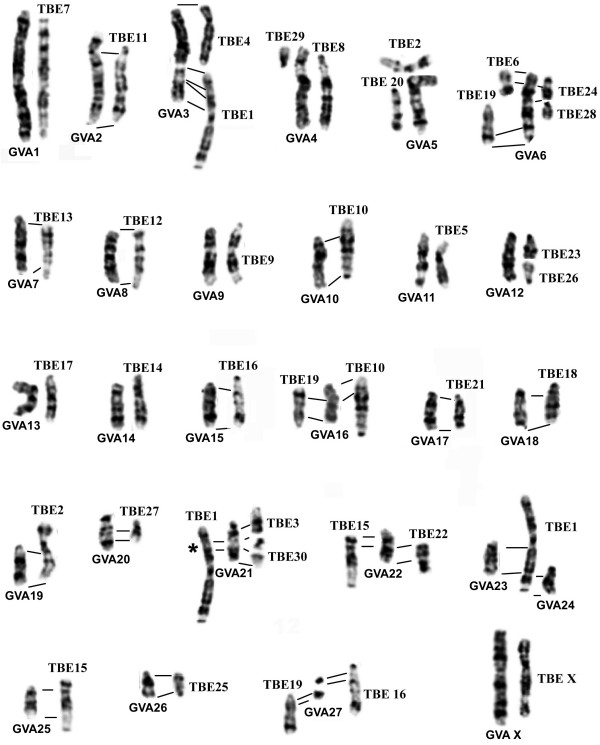
**Correspondence between flying lemur (*G. variegatus*, GVA) and tree shrew (*T. belangeri*, TBE) G-banded chromosomes, based on chromosome painting**. The flying lemur chromosome numbers are given below. The tree shrew chromosome numbers are indicated at the top, right or left. * Müller et al [53] failed to detect the human 7 homologous segment on TBE 1p, but Richard et al [30] reported its presence.

In addition to the new insight into the phylogenetic affinity of Dermoptera within Supraprimates, the establishment of the first G-banded karyotype for *G. variegatus *and the human-*G. variegatus *comparative map, together with the published human-*T. belangeri *comparative map, has provided us with an opportunity to compare the G-banded karyotypes of the *T. belangeri *and *G. variegatus *in detail on the basis of genome-wide homologies defined by chromosome painting (Figure 5). Ignoring the morphological changes due to variations in the amount and distribution of heterochromatin, 12 *G. variegatus *autosomes (GVA1, 2, 7–9, 11, 13, 14, 17, 18, 20 and 26) show one-to-one correspondence with their *T. belangeri *counterparts; six *G. variegatus *autosomes (GVA10, 15, 19, 23–25) each correspond to one *T. belangeri *autosomal segments; nine *G. variegatus *autosomes (GVA3-6, 12, 16, 21, 22 and 27) each correspond to between two and four *T. belangeri *chromosomes or chromosomal segments. The G-banding comparison demonstrates that most homologous chromosomes or chromosomal segments between *T. belangeri *and *G. variegatus *defined by comparative painting also show matching G-banding patterns. It is noteworthy that the acrocentric *G. variegatus *chromosome 1 (= HSA2/21/3), the largest autosome that also carries the HSA2/21 association unique for *G. variegatus *and *T. belangeri*, is conserved entirely. In contrast, *G. variegatus *chromosome 11 (= TBE5) that carried the ancestral placentals syntenic association of HSA16/19 has undergone an inversion as demonstrated by the chromosome painting and banding comparisons. At the current resolution level of G-banding and cross-species chromosome painting, our results suggest that simple chromosomal fissions/fusions are the predominant mechanism that differentiates the karyotypes of *G. variegatus *and *T. belangeri*. However, the conservation in G-banding patterns does not necessarily indicate a close phylogenetic relationship between *G. variegatus *and *T. belangeri*. Although we have found only one interchromosomal rearrangement that unites Scandentia and Dermoptera into one clade, small and cryptic intrachromosomal rearrangements (inversions) will escape detection by the current methods. Further comparative high-resolution gene mapping in flying lemurs, tree shrews as well as outgroup species such as the rabbits, squirrels and prosimians may reveal some intrachromosomal rearrangements that could serve as additional cytogenetic evidence for the proposed Scandentia and Dermoptera sister clade.

## Conclusion

We have characterized the G-banded karyotype of *G. variegatus *and established the first genome-wide comparative chromosome map between human and *G. variegatus *by reciprocal chromosome painting. Comparative analysis of the chromosome painting data from representative species of each order within the superordinal clade Supraprimates provides new molecular cytogenetic evidence that supports a sister-clade relationship between Dermoptera and Scandentia. However, our analysis has failed to identify any cytogenetic signature for well-defined clades such as the Euarchonta and Supraprimates as well as for other controversial clades.

## Methods

### Cell culture, metaphase preparation and G-banding

Fibroblast cell lines derived from two male *G. variegatus *(from Thailand) and one male tree shrew (*Tupaia belangeri*, TBE, 2*n *= 62, KCB 200305) were established in Kunming Cell Bank, the Chinese Academy of Sciences (Kunming, Yunnan, People's Republic of China) and the Paris Natural History Museum (Paris, France) from skin biopsies. Cell culture, metaphase preparations and G-banding were carried out following conventional methods as described previously [31,64]. The chromosomes of *T. belangeri *were numbered according to a previously published G-banded karyotype [53]. The karyotype of *G. variegatus *was arranged according to relative chromosomal size.

### Flow sorting and generation of chromosome-specific painting probes

Chromosome preparation of *G. variegatus *for flow sorting followed the method described previously [26], and was sorted on a dual-laser cell sorter (FACStar Plus, Becton Dickinson) staining with chromomycin A3 (40 μg/ml, Sigma) and Hoechst 33258 (2 μg/ml, Sigma). Chromosome-specific paints for *G. variegatus *were generated from flow-sorted chromosomes by degenerate oligonucleotide primed (DOP) polymerase chain reaction (PCR) amplification [65]. DOP-PCR-amplified chromosome-specific DNAs were labeled with biotin-16-dUTP, FITC-12-dUTP (Roche) or Cy3-dUTP (Amersham) by secondary PCR amplification. Human painting probes used in this study were also prepared from flow-sorted chromosomes as described previously [66].

### FISH, image capture and processing

Reciprocal cross-species chromosome painting between human and *G. variegatus *and post-hybridization detection followed previous procedures [40,66]. Biotin-labeled probes were visualized using a layer of Cy3-avidin (1:1,000 dilution; Amersham). FITC-labeled probes were visualized using rabbit anti-FITC (1:200; DAKO) and goat anti-rabbit-FITC (1:200; Vector Laboratories). After detection, slides were mounted in Vectashield mounting medium with DAPI (4'6-diamidino-2-phenylindole, Vector Laboratories). Digital images were acquired using the CytoVision system (Applied Imaging Corp.) with a CCD camera mounted on a microscope (Olympus or Zeiss). Hybridization signals were assigned to specific chromosomes or chromosome regions defined by DAPI-banding patterns.

## Authors' contributions

WN and BF performed chromosome-painting experiments. PCMO sorted the chromosomes. JW, WS, AT and VV collected the materials, cultured the cells, and prepared the chromosomal suspensions. WN and FY analyzed the data, and wrote the paper. FY and MAFS conceived of the experiment. PCMO and MAFS revised the paper. All of the authors read and approved the final manuscript.
